# Comparison of mortality rate and septic and aseptic revisions in total hip arthroplasties for osteoarthritis and femoral neck fracture: an analysis of the German Arthroplasty Registry

**DOI:** 10.1186/s10195-023-00711-9

**Published:** 2023-06-17

**Authors:** Dominik Szymski, Nike Walter, Paula Krull, Oliver Melsheimer, Melanie Schindler, Alexander Grimberg, Volker Alt, Arnd Steinbrueck, Markus Rupp

**Affiliations:** 1 Department for Trauma Surgery, University Hospital Regensburg, Franz-Josef-Strauss-Allee 11, 93053 Regensburg, Germany; 2 Endoprothesenregister Deutschland gGmbH (EPRD), Berlin, Germany; 3 Orthopädisch Chirurgisches Kompetenzzentrum Augsburg (OCKA), Augsburg, Germany

**Keywords:** Hip arthroplasty, Osteoarthritis, Femoral neck, Fracture, Mortality, Periprosthetic joint infection, Revision, Arthrosis, Degeneration

## Abstract

**Background:**

Indications for total hip arthroplasties (THA) differ from primary osteoarthritis (OA), which allows elective surgery through femoral neck fractures (FNF), which require timely surgical care. The aim of this investigation was to compare mortality and revisions in THA for primary OA and FNF.

**Methods:**

Data collection for this study was performed using the German Arthroplasty Registry (EPRD) with analysis THA for the treatment of FNF and OA. Cases were matched 1:1 according to age, sex, body mass index (BMI), cementation, and the Elixhauser score using Mahalanobis distance matching.

**Results:**

Overall 43,436 cases of THA for the treatment of OA and FNF were analyzed in this study. Mortality was significantly increased in FNF, with 12.6% after 1 year and 36.5% after 5 years compared with 3.0% and 18.7% in OA, respectively (*p* < 0.0001). The proportion for septic and aseptic revisions was significantly increased in FNF (*p* < 0.0001). Main causes for an aseptic failure were mechanical complications (OA: 1.1%; FNF: 2.4%; *p* < 0.0001) and periprosthetic fractures (OA: 0.2%; FNF: 0.4%; *p* = 0.021). As influencing factors for male patients with septic failure (*p* < 0.002), increased BMI and Elixhauser comorbidity score and diagnosis of fracture (all *p* < 0.0001) were identified. For aseptic revision surgeries, BMI, Elixhauser score, and FNF were influencing factors (*p* < 0.0001), while all cemented and hybrid cemented THA were associated with a risk reduction for aseptic failure within 90 days after surgery (*p* < 0.0001).

**Conclusion:**

In femoral neck fractures treated with THA, a significant higher mortality, as well as septic and aseptic failure rate, was demonstrated compared with prosthesis for the therapy of osteoarthritis. Increased Elixhauser comorbidity score and BMI are the main influencing factors for development of septic or aseptic failure and can represent a potential approach for prevention measures.

*Level of evidence*: Level III, Prognostic.

## Introduction

Total hip arthroplasty (THA) is one of the most commonly performed orthopedic procedures worldwide. Based on historical data provided by Organisation for Economic Co-operation and Development (OECD) countries and a projection analysis, in the year 2050 2.8 (2.6–2.9) million THA will be implanted, while in the year 2015 1.8 million procedures were registered. This corresponds to an annual growth rate of 1.2% and, for some countries, a growth rate of over 125% between 2015 and 2050 [1]. Several indications are responsible for the implantation of THA. Two of the most common are osteoarthritis (OA) and femoral neck fracture (FNF) [2]. Due to the different circumstances, both procedures face different risk profiles. For one thing, THA for the treatment of OA allows patient optimization, while FNF requires timely surgical care, which does not allow optimization of comorbidities and infection-prevention strategies, as is the case in elective joint arthroplasty [3]. In a meta-analysis, Berstock et al. reported a mortality rate after THA for OA of 0.65% (95% CI 0.50–0.81) within the first 90 days [4]. Patients receiving a THA for fracture treatment had a multiple-fold increase in mortality rate, with up to 24% after 1 year [5].

The main reasons for required revision surgery are periprosthetic joint infections (PJI), dislocations, periprosthetic fractures, and mechanical loosening [6]. For PJIs Blomfeld et al. described in a single-center cohort study an increased hazards ratio of 4.3 after FNF compared with osteoarthritis for the development of an infection [7]. The rates for infections for THA were reported to be around 1% annually following OA and up to 10% following femoral neck fracture [8].

The aim of the present investigation was (1) to report the rate of mortality for THA following primary OA and FNF and (2) to determine the rate of septic and aseptic revisions after treatment of primary OA and FNF with THA. (3) Risk factors for the occurrence of aseptic and septic failure of THA should be identified and compared between THA for OA or FNF, and analyzed and compared between THA for the treatment of FNF and OA.

## Material and methods

### Data collection

The investigation is based on the data set of the German Arthroplasty Registry [*Endoprothesenregister Deutschland* (EPRD). Through collaboration with statutory health insurance funds (AOK Bundesverband GbR, Verband der Ersatzkassen e.V vdek), the German Medical Technology Association (BVMed), and several participating hospitals, approximately 70% of all hip and knee arthroplasties performed in Germany were covered in the registry by 2020 [9]. Data of the two participating health insurance associations (AOK-B, vdek) cover 65% of the German population and are cross-validated by data input of surgeons. If revision surgeries were performed in a non-participating hospital, a follow-up was performed based on insurance billing data. With the exception of procedures performed outside of Germany, this algorithm ensures near perfect tracking of patients insured by these companies [10]. The German versions of the International Classification of Procedures in Medicine (ICPM), the Operation and Procedure Code (OPS) 301 system, and the 10th International Classification of Diseases (ICD-10) were used for registration of diagnosis and procedures.

### Patients

**Fig. 1:**
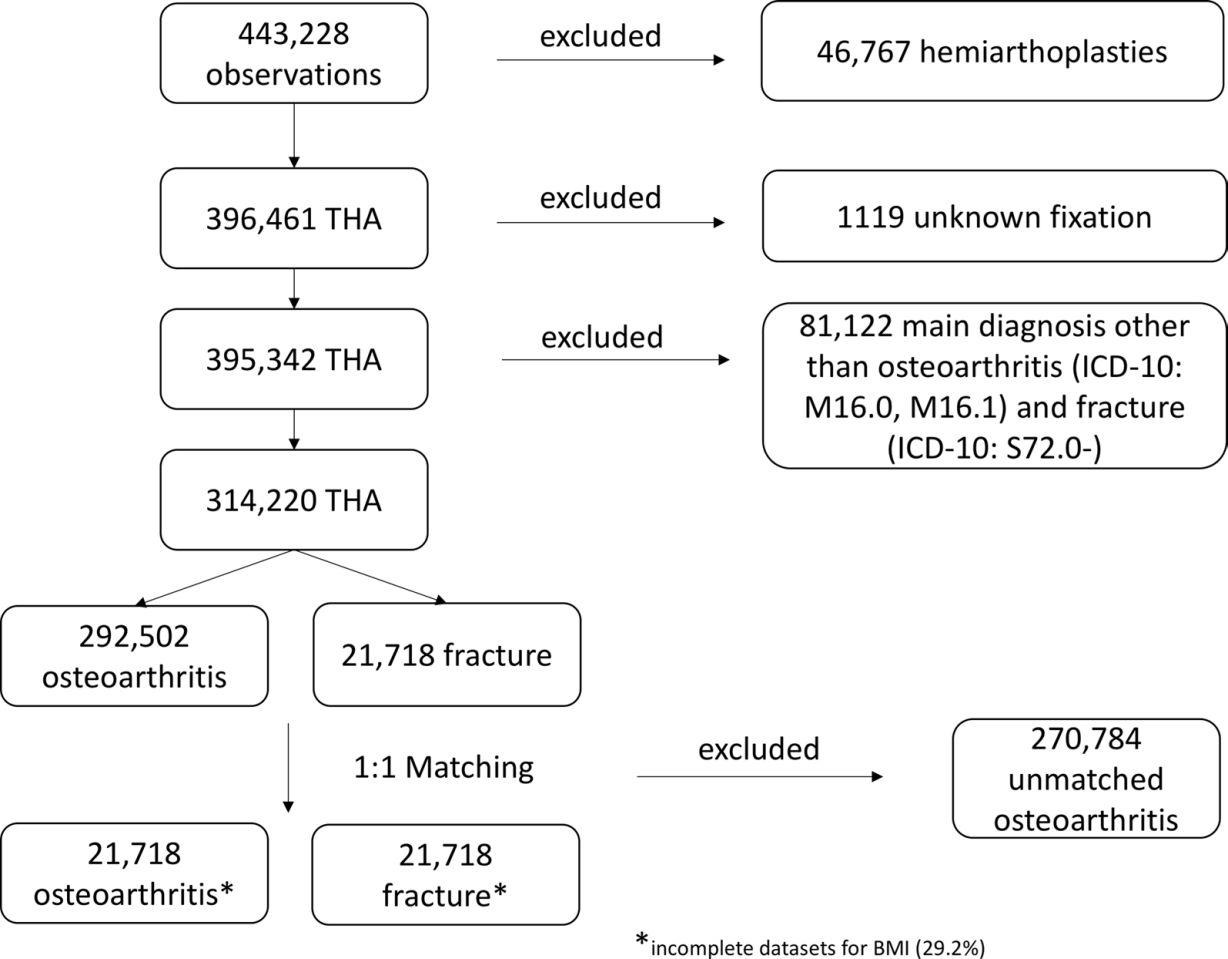
Flowchart of the study collective with inclusion and exclusion criteria

Patients with THA for the treatment of primary osteoarthritis (ICD-10: M16.0 and M16.1) and after femoral neck fracture as main diagnosis (ICD-10: S72.0–) between 1 January 2013 and 3 December 2022 were included in the present analysis of the German Arthroplasty Registry (EPRD). For the analysis patients were divided into subpopulations according to the reason for THA implantation. Patients with THA for the treatment of primary OA and for the treatment of FNF were matched according to age, sex, body mass index (BMI), cementation technique, and Elixhauser comorbidity score (in the van Walraven variant) using Mahalanobis distance matching in a 1:1 ratio. The Elixhauser score is an index that pools a variety of comorbidities of different organ systems and entities [11]. Mortality after implantation was determined by matching arthroplasty data with deaths recorded in insurance data. In addition to comorbidities, all other billing diagnoses are recorded in the arthroplasty registry and used to determine influencing factors. Failure of THA was determined by analysis of reasons for revision through a search of the ICD-10 code (ICD-10: T84.–) during revision surgery in the registry and registration by surgeons during the data input of arthroplasty register. According to European Bone and Joint Infection Society (EBJIS) guidelines, a definition of periprosthetic joint infection was obtained by surgeons and coded as PJI and therefore registered as septic failure in the registry [12]. All failures of THA not matching the EBJIS criteria for PJI were categorized as aseptic failure. Through analysis of surgical indication code by ICD-10 and through registration of the surgeons of the cause for revision in the Arthroplasty Registry, all aseptic revisions were classified according to reason for revision. Exclusion criteria were patients with unclear main diagnoses or implantation of total hip arthroplasty following a reason other than FNF or primary OA of the hip. Patients with unknown fixation of components or type of prosthesis were also excluded from the data collection (Fig. 1).

### Statistical analysis

To determine the mortality and revision rate and timeframe of septic and aseptic revision in THA after OA and FNF, data from the German Arthroplasty Registry was analyzed according to revision surgeries following THA implantation. To account for bias in the selection of OA and FNF patients, matching of cases was performed using the variables of sex, age at the time of surgery, fixation method, and the van Walraven weighted version of the Elixhauser index [11] and, if the information was available, the patient’s BMI. The statistical program R (R Foundation for Statistical Computing version 4.2.0, Vienna, Austria) was used to perform the statistical analysis. For postmatching statistical analysis, cumulative incidences were calculated with Kaplan–Meier estimates, and hazard ratios were calculated by using a Cox proportional-hazards model [13]. Log-rank test was used for the comparison of THA for the treatment of FNF and THA for the therapy of OA. Categorical variables are presented in number of observations and frequency, and continuous variables in mean and standard deviation. Significance level was set at alpha = 0.05.

## Results

Overall, 357,661 patients with THA for the treatment of primary OA and for the treatment of FNF were identified in the EPRD. After exclusion of incomplete datasets and 1:1 matching using age at admission, sex, BMI, Elixhauser comorbidity score, and fixation method, 43,436 cases were included into further data analysis (OA: 21,718 patients; FNF: 21,718 patients). BMI and Elixhauser score remain statistically significant after matching; however, balance was satisfactorily improved (standardized mean difference was below 0.05 and variance below 1.10 for both variables; Table 1).

**Table 1:** Anthropometry and risk factors after matching (1:1) of total hip arthroplasties for the treatment of primary osteoarthritis and femoral neck fracture

	Osteoarthritis (OA)	Intracapsular femoral neck fracture (FNF)	*p*-Value
Number (*n*)	21,718	21,718	
Age (years)	74.8 ± 9.52	74.7 ± 9.81	*p* = 0.331
Sex (female) *n* (%)	15,206 (70.0)	15,186 (69.9)	*p* = 0.842
Elixhauser comorbidity score	5.08 ± 6.56	5.24 ± 6.79	*p* = 0.014
Body mass index (BMI) in kg/m^2^	25.5 ± 4.25	25.1 ± 4.35	*p* < 0.0001
Cementless fixation (%)	10,209 (47.0)	10,182 (46.9)	*p* = 0.803

BMI and Elixhauser score remain statistically significant after matching; however, balance is satisfactorily improved (standardized mean difference is below 0.05 and variance below 1.10 for both variables)

**Fig. 2:**
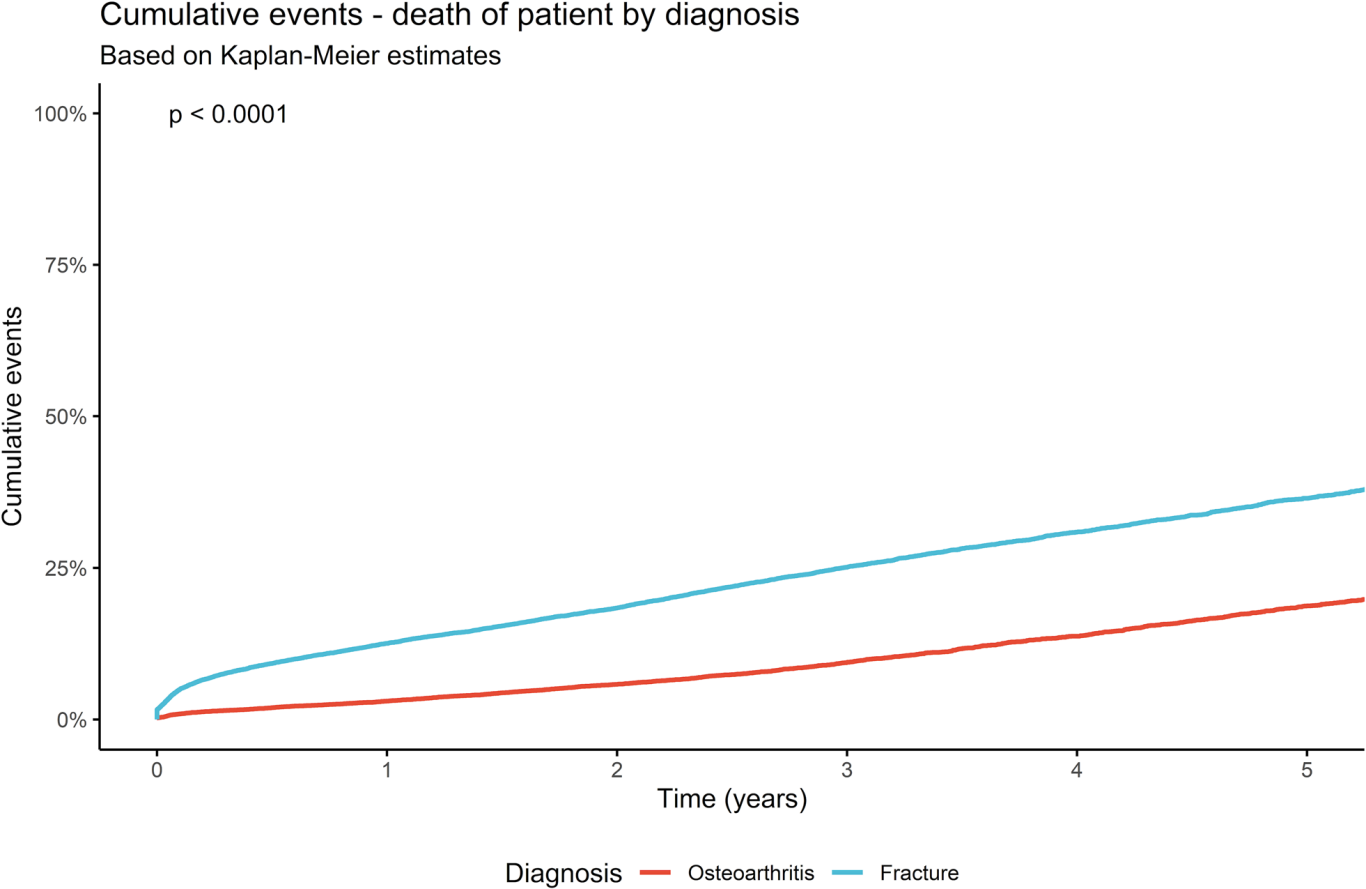
Mortality rate in total hip arthroplasties for the treatment of primary osteoarthritis and after femoral neck fractures in a period of 5 years (see Table 3 for corresponding 95% confidence intervals)

The mortality rate after THA increased from 0.8% after 1 month to 2.0% after 6 months followed by 3.0% after 1 year and 18.7% after 5 years in cases of hip replacement for the treatment of OA. After FNF the rate of mortality increased from 4.6% after 1 month to 9.2% after 6 months and to 12.6% after 1 year and 36.5% 5 years after implantation of a THA (*p* < 0.0001; Fig. 2, Table 3).

**Fig. 3:**
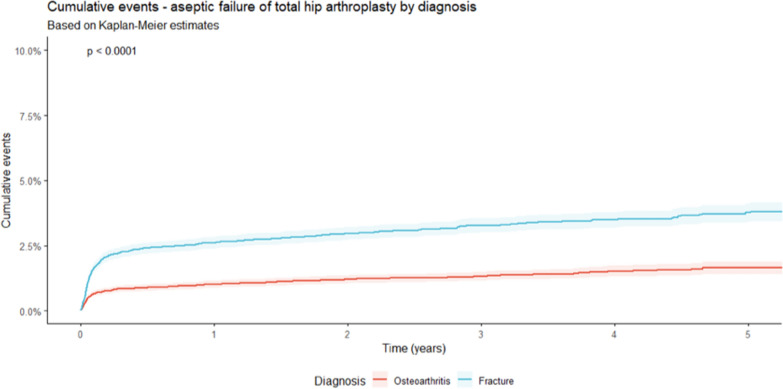
Development of aseptic failures in total hip arthroplasties for the treatment of primary osteoarthritis and after femoral neck fractures in a period of 5 years (see Table 3 for corresponding 95% confidence intervals)

**Fig. 4:**
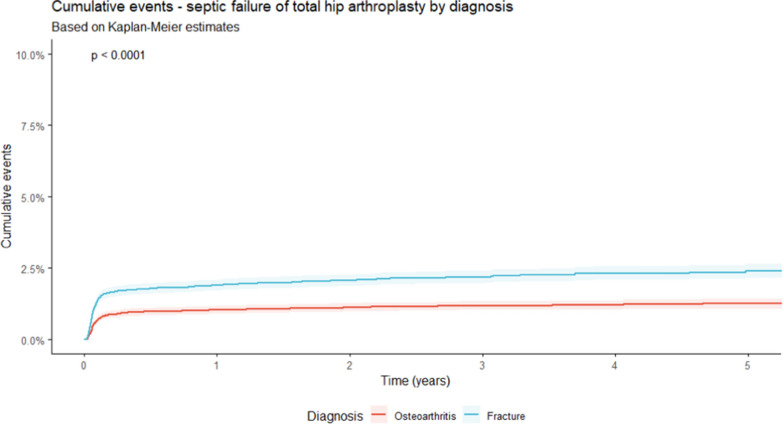
Development of septic failures in total hip arthroplasties for the treatment of primary osteoarthritis and after femoral neck fractures in a period of 5 years (see Table 3 for corresponding 95% confidence intervals)

Within a 5-year timeframe, a significantly higher rate of aseptic failure was detected for patients treated with a THA after FNF compared with THA for OA (*p* < 0.0001; Fig. 3). After FNF an aseptic failure of 2.9% was reported between implantation and revision surgery after a mean period of 194 days. For cases after OA, 1.3% of failure after a mean period of 265 days was detected. The main reasons for aseptic revision surgery in both subgroups were mechanical complications (OA: 1.1%; FNF: 2.4%; *p* < 0.0001) and periprosthetic fractures (OA: 0.2%; FNF: 0.4%; *p* < 0.021; Table 2). Thereby, the rate of infection raised from 1.2% after 1 month to 1.9% after 1 year and 2.4% after 5 years in THA after fracture, while in cases with treatment of OA, 0.6%, 1.0%, and 1.3% were reported during the same time (Fig. 4, Table 3). As influencing factors in septic revisions, male patients [hazards ratio (HR) = 1.342 (95% CI 1.110–1.622); *p* = 0.002], BMI [HR = 1.090 (95% CI 1.071–1.108); *p* < 0.0001], the diagnosis of a femoral neck fracture [HR = 2.028 (95% CI 1.672–2.461); *p* < 0.0001], and within the first 90 days after implementation the Elixhauser comorbidity score [HR = 1.063 (95% CI 1.051–1.076); *p* < 0.0001] were identified. For aseptic failure, BMI [HR = 1.028 (95% CI 1.012–1.044); *p* < 0.001], Elixhauser score [HR = 1.037 (95% CI 1.027–1.047); *p* < 0.0001], and the diagnosis of a fracture [HR = 2.637 (95% CI 2.250–3.091); *p* < 0.0001] were identified as influencing factors for aseptic failure with a Cox proportional-hazards model. As protective factors, all kinds of cemented stem fixation (cemented and hybrid cemented) were detected. The protective effect for aseptic failure was particularly evident within the first 90 days after implantation [all cemented: HR = 0.351 (95% CI 0.258–0.477); *p* < 0.0001; hybrid cemented: HR = 0.618 (95% CI 0.509–0.751); *p* < 0.0001].

**Table 2:** Septic and aseptic failure of patients after total hip arthroplasty for the treatment of primary osteoarthritis and femoral neck fracture

	Osteoarthritis (OA)	Intracapsular femoral neck fracture (FNF)	*p*-Value
Number of cases (*n*)	21,718	21,718	*p* = 1
Mean time to failure (any reason) in days ± SD	1010 ± 669	833 ± 670	*p* < 0.0001
Reason for aseptic failure			
- Mechanical complication	232 (1.1)	522 (2.4)	*p* < 0.0001
- Periprosthetic fracture	50 (0.2)	77 (0.4)	*p* < 0.021
- Dislocation	13 (0.1)	57 (0.3)	*p* < 0.0001
Mean time to aseptic failure in days ± SD	265 ± 460	194 ± 384	*p* = 0.015
Septic failure	244 (1.1)	425 (2.0)	*p* < 0.0001
Mean time to septic failure in days ± SD	123 ± 267	125 ± 280	*p* = 0.922
Death of patient associated with septic failure	57 (0.3)	136 (0.6)	*p* < 0.0001
Death of patient for any reason	2212 (10.2)	5046 (23.2)	*p* < 0.0001

**Table 3:** Mortality and failure rate for aseptic and septic reasons for total hip arthroplasty after primary osteoarthritis and femoral neck fracture with corresponding 95% confidence intervals

		1 month	2 months	3 months	6 months	1 year	3 years	5 years
Death of patient Cumulative events (%) (95% confidence interval)	Osteoarthritis	0.8 (0.7–1.0)	1.2 (1.0–1.3)	1.4 (1.2–1.6)	2.0 (1.8–2.1)	3.0 (2.8–3.3)	9.4 (8.9–9.9)	18.7 (17.8–19.7)
	Femoral neck fracture	4.6 (4.3–4.8)	6.1 (5.8–6.4)	7.1 (6.8–7.5)	9.2 (8.9–9.6)	12.6 (12.1–13.0)	25.1 (24.4–25.8)	36.5 (35.5–37.5)
Aseptic failure Cumulative events (%) (95% confidence interval)	Osteoarthritis	0.6 (0.5–0.7)	0.7 (0.6–0.9)	0.8 (0.7–0.9)	0.9 (0.8–1.0)	1.0 (0.9–1.2)	1.3 (1.2–1.5)	1.7 (1.4–1.9)
	Femoral neck fracture	1.5 (1.3–1.6)	2.0 (1.8–2.2)	2.2 (2.0–2.4)	2.4 (2.2–2.6)	2.6 (2.4–2.8)	3.3 (3.0–3.6)	3.8 (3.4–4.1)
Septic failure Cumulative events (%) (95% confidence interval)	Osteoarthritis	0.6 (0.5–0.7)	0.9 (0.7–1.0)	0.9 (0.8–1.0)	1.0 (0.8–1.1)	1.0 (0.9–1.2)	1.2 (1.0–1.3)	1.3 (1.1–1.4)
	Femoral neck fracture	1.2 (1.1–1.4)	1.6 (1.4–1.8)	1.7 (1.5–1.9)	1.8 (1.6–2.0)	1.9 (1.7–2.1)	2.2 (2.0–2.4)	2.4 (2.1–2.7)

## Discussion

In this analysis 43,436 matched patients with THA for the treatment of primary OA or FNF registered in the German Arthroplasty Registry (EPRD) were included. For patients treated with a THA after FNF, a significantly increased mortality, as well aseptic and septic failure rate, was detected. In particular, patients who were male, had FNF as the reason for THA implantation, and had an increased BMI and Elixhauser comorbidity score were associated with a higher rate of THA failure. All cemented and hybrid cemented THA, however, demonstrated a reduction of failure in aseptic cases.

Le Manach et al. demonstrated a significantly increased rate for the in-hospital mortality after implantation of THA for FNF. In their analysis of the French Discharge Database, a relative risk (RR) of 5.88 compared with elective hip arthroplasties was reported [14]. The largest amount of available data on mortality rate after fracture treatment was published by Gundel et al., reporting 15 years of Danish register data. The mortality rate raised from 9.6% 30 days after surgery to 16% at 90 days after implantation and 27% after 1 year [15]. A mortality rate of up to 36.7% in the third year after implantation was reported [5, 16]. Comparing these results to THA after OA already at 90 days post-implantation, a reduced rate of mortality (0.56–0.65%) was described in literature [4, 17]. Long-term results also demonstrated a reduced rate, with 29.5% mortality 10 years after joint replacement [18]. The present results demonstrate a pattern reporting similar to the data of the German Arthroplasty Register (EPRD). After 5 years the mortality in FNF, with 36.5%, was almost double that of OA (18.7%; *p* < 0.0001). Similar results were also reported by Charette et al. and Sassoon et al., comparing fracture and osteoarthritis patients after the implantation of a THA [19, 20]. The increased mortality in THA is associated with the occurrence of multiple factors. Regardless of the underlying disease, which was the reason for THA implantation, increased age, BMI, and number and severity of comorbidities are in general decisive factors [5, 16]. While often bone cement implantation syndrome (BCIS) is under suspicion in relation to increased mortality rates, no significant differences between cemented and cementless hemiarthroplasties were reported in a previous investigation [21].

In OA the surgical treatment can be prepared over weeks through limitation and treatment of risk factors for failure; in the fracture situation, a timely implantation is required to achieve a reduction of mortality [5]. Cardiopulmonary diseases, endocrinological diseases, and body weight can be improved in the preoperative setting, but often takes several weeks or even months to achieve success [22, 23]. The dilemma between scheduled and unscheduled surgeries continues when it comes to the skin preparation before intervention. Planned THA patients are often treated with a self-application wash kit to reduce dermal bacterial colonization, which is only barley possible or performed in the emergency setting [24].

These aforementioned issues with an unplanned surgery make all kinds of failure more likely (Odds ratio: 2.8; 95% CI 2.1–3.8) [25]. In particular septic failures are more likely [26]. Patient-specific factors are thereby in a complex interaction with surgical specifications, as the implant and technique, and are thus involved in the development of an infection [27]. Oltean-Dan et al. therefore reported a particularly higher risk of FNF patients developing PJI [28]. Also other publications referring to a comparison of FNF and OA patients reported a significantly increased PJI rate for fracture patients [19, 20, 25]. In our investigation patients after fracture showed a significantly increased rate of septic failure in the log-rank test (*p* < 0.0001). While in our OA population after 6 months 1.0%, after 1 year 1.0% and after 5 years 1.3% suffered from PJI, in the fracture subgroup after an equal period of time 1.8%, 1.9%, and 2.4% needed a septic revision surgery, respectively. In a meta-analysis Ren et al. reported FNF as one of the major risk factors for the development of a PJI with a risk ratio (RR) of 1.75 (95% CI 1.39–2.20). Hence FNF was demonstrated in this meta-analysis to be of significant influence on the development of infection next to BMI (RR: 2.40), diabetes mellitus (RR: 1.64), avascular necrosis of the femoral head (RR: 1.64), rheumatoid arthritis (RR: 1.37), and cardiovascular diseases (RR: 1.34). However Ren et al. demonstrated OA to be a preventive factor, with a risk ratio of 0.70 (95% CI 0.62–0.79) [29]. Additionally, our present analysis of the German Arthroplasty Registry demonstrates male sex, BMI, and FNF to be relevant factors for the occurrence of PJI after THA. Even after treatment of PJI and revision surgery, patients with an FNF as underlying diagnosis demonstrated a significantly increased risk for re-infection (*p* < 0.05) in the recent literature [30]. Interestingly the mean time between implantation and septic failure remains equal, with 123 ± 267 (OA) and 125 ± 280 (FNF) days (*p* < 0.922) in our analysis.

In contrast, when considering aseptic revisions, a significantly shorter time falls between implantation and time of revision in FNF cases (*p* = 0.015). In the present analysis, the rate for aseptic revision was significantly increased in patients with FNF compared with patients with OA (*p* < 0.0001). In both entities, mechanical complications and periprosthetic fractures were the main reasons for a performed revision surgery in aseptic cases. These results are comparable to recently published literature, where a significantly increased rate of any complication was reported in THA for the treatment of an FNF [14, 20, 25, 31]. Mechanical and biological factors play an important role in the development of osteolysis following a THA and consequently in the development of aseptic loosening and periprosthetic fractures. Inflammatory processes are not only relevant in the primary healing process but have been also discussed as important in periprosthetic osteolysis [32]. The significantly increased rate of aseptic complications after fracture can potentially be explained by the triggered immune reaction, which takes place between the injury and the treatment, in the case of a fracture compared with planned arthroplasties for OA [32, 33]. Oltean-Dan et al. demonstrated a shorter time period between implantation and revision surgery in FNF cases. Similarly, the most common reasons were aseptic loosening (37%) and periprosthetic fractures (23%) [28]. The most likely reason for aspetic revision in the present analysis were mechanical complications, with a significantly higher proportion in fracture treatment (2.4% versus 1.1%; *p* < 0.0001). In the literature aseptic loosening (51.9%) is the most commonly reported cause for a revision surgery for THA after OA, followed by PJI (15.6%) [31]. Analyzing influencing factors on aseptic revisions through a Cox proportional-hazards model, a similar pattern as in septic revisions was noticed. The diagnosis of a fracture (HR: 2.637), Elixhauser comorbidity score (HR: 1.037), and BMI (HR: 1.028) are thereby factors that increase the risk of aseptic revision surgery. Interestingly, cemented THA demonstrated a protective influence for aseptic revision within the first 90 days after implantation. Both totally cemented (HR: 0.351) and hybrid cemented (HR: 0.618) THA reduce the risk for required aseptic revisions. Two systemic reviews and meta-analyses by Prokopetz et al. and Cherian et al. revealed several influencing factors for aseptic failure, such as age, sex, comorbidities, surgical volume, and reason for arthroplasty [26, 34]. For aseptic loosening, a higher risk was reported in male patients of younger age [26, 34].

The increased mortality and proportion of septic and aseptic revisions after THA for the treatment of FNF is of great clinical importance. The complication rate after THA for OA is the baseline for a population with optimal preparation before arthroplasty. After fracture, however, a fast time between accident and surgery is necessary to reduce the mortality [5]. The modifiable risk factors found in our investigation are possible approaches for implementation of prevention strategies and patient optimization in the preoperative setting when THA for FNF is necessary. Introduction of a standardized pre- and perioperative protocol with elements of known pre-habilitation measures, such as the use of preoperative wash kits, adjustment of a regular blood glucose level, and strict peri- and postoperative surveillance, can help improve the mortality and failure rate after FNF [3, 35, 36]. However, some elements of pre-habilitation, such as regular physiotherapy before hip arthroplasty to improve strength and mobility, are not feasible in fracture patients [36]. The Center for Disease Control and Prevention (CDC) reports diabetes, smoking, and coexisting infection as relevant comorbidities for PJI with recommendation of improvement [3]. Even in patients without the diagnosis of diabetes mellitus, but with an increased blood glucose value pre-, peri-, and postoperatively had a significantly higher rate of infections following lower limb arthroplasties [37]. To our knowledge right now there are no guidelines on the management of blood glucose levels, and further research on this issue is necessary. The use of preoperative wash-kits with chlorhexidine or soap demonstrated a significant risk reduction in patients with arthroplasties compared with non-compliance of cleaning instructions, with a odds ratio of 0.27 (95% CI 0.09–0.79) [38].

The present study inherits several limitations that should be mentioned. Despite multiple advantages of the German Arthroplasty Registry having a huge database on THAs, some limitations were noticed due to the conceptualization of the study. Because of the data registration through registration by surgeons, the quality of data is dependent on the correct coding of diagnoses and procedures. To minimize this effect and limitation of the study design, included patient data regarding THA were cross-validated by using insurance data. For a more detailed evaluation of risk factors and their influence, further investigations compared with a non-fracture and non-osteoarthritis population is necessary. Another limitation is caused by the age of the registry, which does not permit the investigation of a follow-up longer than 5 years at the moment. The percentage of uncemented and cemented THA does not reflect the actual status in the world. To answer the research question of differences in mortality rate, as well as aseptic and septic revision rate, the populations with THA for primary OA and FNF were also matched with regard to fixation, and a balanced share of uncemented and cemented fixation was chosen.

## Conclusion

Patients with FNF treated with a THA demonstrated an increased rate of mortality, as well as enhanced proportion for septic and aseptic failure. Femoral neck fracture, an increased Elixhauser comorbidity score and obesity are the major influencing factors for development of septic or aseptic failure and can represent a potential approach for prevention measures. Optimization of these factors through standardized perioperative procedures and the use of cemented THA in patients with risk factors for aseptic revision surgery may lead to a reduction of failure risk.

## Data Availability

On request.
